# Parameter estimation of dynamic biological network models using integrated fluxes

**DOI:** 10.1186/s12918-014-0127-x

**Published:** 2014-11-18

**Authors:** Yang Liu, Rudiyanto Gunawan

**Affiliations:** Institute for Chemical and Bioengineering, ETH Zurich, Vladimir-Perlog-Weg 1, Zurich, 8093 Switzerland

**Keywords:** Parameter estimation, ODE model, Power-law model, Lin-log model

## Abstract

**Background:**

Parameter estimation is often the bottlenecking step in biological system modeling. For ordinary differential equation (ODE) models, the challenge in this estimation has been attributed to not only the lack of parameter identifiability, but also computational issues such as finding globally optimal parameter estimates over highly multidimensional search space. Recent methods using incremental estimation approach could alleviate the computational difficulty by performing the parameter estimation one-reaction-at-a-time. However, incremental estimation strategies usually require data smoothing and are known to produce biased parameter estimates.

**Results:**

In this article, we presented a new parameter estimation method called integrated flux parameter estimation (IFPE). We employed the integral form of the ODE such that we could compute the integral of reaction fluxes from time-series concentration data without data smoothing. Here, we formulated the parameter estimation as a nested optimization problem. In the outer optimization, we performed a minimization of model prediction errors over parameters associated with a subset of reactions labeled as independent. The dimension of the independent reaction subset was equal to the degrees of freedom in the calculation of integrated fluxes (IF) from concentration data. We selected the independent reactions such that given their IF values, the IFs of the remaining (dependent) reactions could be uniquely determined. Meanwhile, in the inner optimization, we estimated the model parameters associated with the dependent reactions, one-reaction-at-a-time, by minimizing the dependent IF prediction errors. We demonstrated the performance of the IFPE method using two case studies: a generalized mass action model of a branched pathway and a lin-log ODE model of *Lactococcus lactis* glycolytic pathway.

**Conclusions:**

The IFPE significantly outperformed standard simultaneous parameter estimation in terms of computational efficiency and scaling. In comparison to incremental parameter estimation (IPE) method, the IFPE produced parameter estimates with significantly lower bias and did not require time-series data smoothing. The advantages of IFPE over the IPE however came at the cost of a small increase in the computational time.

**Electronic supplementary material:**

The online version of this article (doi:10.1186/s12918-014-0127-x) contains supplementary material, which is available to authorized users.

## Background

Mathematical modeling is one of the pillars of systems biology. Here, ODEs have been commonly used to model cellular systems, especially when dynamical behavior is of interest. An ODE model is formulated based on viewing cellular networks as chemical reaction networks, where the equations describe the mass or molar balance as follow:

(1) $$ \frac{d\mathbf{X}(t)}{dt}=\mathbf{S}\mathbf{v}(\mathbf{X}(t),\mathbf{p});\ \ \ \mathbf{X}(0)=\mathbf{X}_{0},  $$

where **X**(*t*)∈**R**^*m*^ is the vector of *m* species concentrations, **S**∈**R**^*m*×*n*^ is the stoichiometric matrix, **v**(**X(t)**,**p**) is the vector of *n* reaction rate equations, **p**∈**R**^*d*^ is the vector of *d* kinetic parameters, and **X**_0_ is the vector of initial concentrations. The creation of ODE models in biology is often hampered by imprecise knowledge of the reaction rate equations and kinetic parameters [1]. Therefore, many model parameters have to be estimated from experimental data. Intuitively, time-series concentration data are desirable for estimating kinetic parameters of ODE models.

Model parameter estimation is typically formulated as a global optimization problem, minimizing the difference between experimental observations and model prediction. We can classify existing methods for estimating ODE model parameters into two general groups: simultaneous and incremental approach [2]. In the simultaneous approach, we search for the optimal parameter combination that minimizes the deviation of simulated concentration predictions from the experimental concentration data. Unfortunately, the parameter estimation from biological data is often underdetermined, where many parameter combinations could fit the data equally well [3,4]. In addition, other computational factors such as finding the global optimal solution over highly multidimensional parameter space and integrating stiff ODEs, often make the parameter estimation numerically intractable, even for models with 10–20 parameters [5].

The incremental approach has been used recently to alleviate the computational issues mentioned above [6-9]. In this approach, the parameter estimation is performed in several (incremental) steps. First, we smoothen time-series concentration data **X**_*M*_(*t*), and differentiate the resulting smoothing functions to obtain estimates of *d***X**_*M*_(*t*)/*dt*. Subsequently, we evaluate the dynamic reaction rate or flux values **v**(*t*) from *d***X**_*M*_(*t*)/*dt* by solving Eq. (1) algebraically. If the stoichiometric matrix **S** has a full column rank, then the flux estimates could be obtained by multiplying the (pseudo-)inverse of **S** with *d***X**_*M*_(*t*)/*dt*. Finally, we perform the kinetic parameter estimation one reaction at a time, by minimizing the sum of squares of the differences between **v**(*t*) and **v**(**X**_*M*_(*t*),**p**). Here, not only the ODE model is not integrated, but also the optimizations involve much smaller parameter space than those in the simultaneous approach. For these reasons, methods based on the incremental approach are usually much faster than those using the simultaneous approach. However, the parameters obtained using incremental estimation strategy are known to be biased and sensitive to data smoothing procedure [2].

In the models of cellular networks, such as metabolic networks, the (pseudo-)inverse of **S** often does not exist since cellular species typically participate in more than one reaction (i.e. *m*<*n*). In addition, experimental measurements are typically taken for only a fraction of the species in the model. In this case, there are degrees of freedom in the flux estimation when applying the incremental estimation approach. Recently, we presented an incremental parameter estimation method that addressed the above issue [10]. We formulated the parameter estimation as a nested optimization problem. Here, we partitioned the reaction rates into independent and dependent subsets, **v**_*I*_ and **v**_*D*_, respectively $\left (\text {i.e}.\ \mathbf {v}=\left [\mathbf {v}_{I}^{T} \mathbf {v}_{D}^{T}\right ]^{T}\right)$. Because of the degrees of freedom, we could select an appropriate **v**_*I*_(*t*), such that **v**_*D*_(*t*) can be uniquely obtained from *d***X**_*M*_(*t*)/*dt* and **v**_*I*_(*t*) using Eq. (1). We formulated the outer optimization problem to estimate parameters associated with the independent reactions, referred to as independent parameters **p**_*I*_. Meanwhile, the remaining (dependent) parameters **p**_*D*_ were estimated in the inner optimization using the dependent reaction flux estimates. Henceforth, we refer the aforementioned estimation method as the incremental parameter estimation (IPE). The IPE could offer several orders of magnitude reduction in the computational time in comparison to standard simultaneous and incremental methods. However, the IPE method was affected by the same issues related to parameter bias and sensitivity to data smoothing mentioned above.

A new class of incremental parameter estimation methods has recently been proposed without the need to smoothen and differentiate noisy time series data [11-13]. In these methods, one calculates the overall extents of reactions directly from time-series concentration data. The extent of a reaction gives the cumulative amount of moles produced by the reaction [11]. In contrast to the traditional incremental estimation strategy, the kinetic parameters are estimated from the reaction extents. However, this method again requires that the stoichiometric matrix **S** has a full column rank.

In this work, we present a new parameter estimation method, called integrated flux parameter estimation, which does not require the stoichiometric matrix **S** to have a full column rank. Similar to the IPE, we formulate the IFPE as a nested optimization problem. However, in contrast to the IPE, the IFPE relies on the calculation of integrated fluxes directly from concentration data, thereby avoiding time-series data smoothing and differentiation. We show using two case studies that the IFPE method can provide much reduced parameter bias and higher reliability in comparison to the IPE method, with only a small increase in the computational time. Nevertheless, for certain ODE models such as those using lin-log rate equations, the IFPE can converge to the optimal parameter solution much faster than the IPE method.

## Methods

In developing the IFPE method, we start with the integral form of the ODE model, given by:

(2) $$ \mathbf{X}(t)-\mathbf{X}(0) = \mathbf{S}{\int_{0}^{t}}\mathbf{v}(\mathbf{X}(\tau),\mathbf{p})d\tau = \mathbf{S}\mathbf{\eta}(\mathbf{X},\mathbf{p})  $$

where **η**(**X**,**p**) denotes the vector of IFs. Here, the *i*-th IF *η*_*i*_ is analogous to the overall extent of the *i*-th reaction per unit volume of a batch reactor [13]. If the stoichiometric matrix **S** has a full column rank, the IF vector can be obtained directly from the concentration measurements **X**_*M*_ as follows:

(3) $$ \mathbf{\eta}(t_{k})=\mathbf{\mathbf{S}^{\dagger}(X}_{M}(t_{k})-\mathbf{X}_{M}(0))  $$

where *t*_*k*_ denotes the *k*-th measurement time point, and **S**^*†*^=**S**^−1^ for a square **S** matrix or $\mathbf {S}^{\dagger }=\left (\mathbf {S}^{T}\mathbf {S}\right)^{-1}\mathbf {S}^{T}$ otherwise.

As mentioned earlier, the matrix **S** in cellular network models often does not have a full column rank. Here, there exist degrees of freedom in the calculation of **η**(*t*_*k*_) from **X**_*M*_, which is equal to *n*− rank(**S**), the dimension of the (right) null space of **S**. In this case, we can select a subset of (independent) reaction rates such that given their IF values $\mathbf {\eta }_{I} \in \mathbf {R}^{n-\operatorname {rank}(\mathbf {S})}$, the values of the remaining (dependent) IFs $\mathbf {\eta }_{D} \in \mathbf {R}^{\operatorname {rank}(\mathbf {S})}$ can be uniquely determined from the relationship in Eq. (2). By partitioning the vector **η** into the independent and dependent components $\mathbf {\eta }=\left [\mathbf {\eta }_{I}^{T} ~~ \mathbf {\eta }_{D}^{T}\right ]^{T}$ and respectively the matrix **S** into **S**=[**S**_*I*_**S**_*D*_], we derive the following relationship between **η**_*I*_ and **η**_*D*_:

(4) $$ \mathbf{\eta}_{D}(t_{k})=\mathbf{S}_{D}^{\dagger}(\mathbf{X}_{M}(t_{k})-\mathbf{X}_{M}(0)-\mathbf{S}_{I}\mathbf{\eta}_{I,k}(\mathbf{X}_{M}, \mathbf{p}_{I})),  $$

where

(5) $$ \mathbf{\eta}_{I,k}(\mathbf{X}_{M},\mathbf{p}_{I})=\int_{0}^{t_{k}}\mathbf{v}_{I}(\mathbf{X}_{M},\mathbf{p}_{I})dt  $$

and **p**_*I*_ are the parameters that appear in the independent reaction subset. Note that when **S** has a full row rank, we can choose **η**_*I*_ such that the submatrix **S**_*D*_ is a square non-singular matrix.

**Figure 1:**
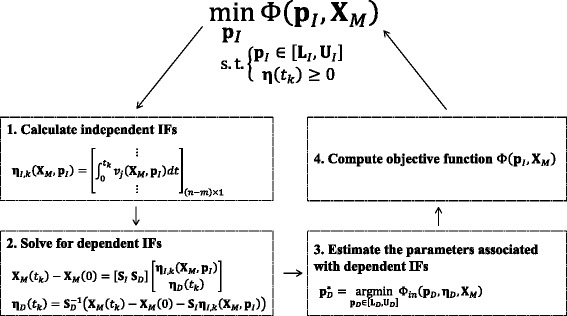
**Flowchart of integrated flux parameter estimation (IFPE).**

Figure 1 shows the schematic diagram of the IFPE method. Here, we consider the scenario where time-series concentration data of all species in the model are available. However, the IFPE can be extended to a more general scenario where only a subset of species are measured (see Additional file 1: Figure S1). In the IFPE, the parameter estimation comprises a nested optimization problem, where the outer optimization involves the minimization of the error function *Φ*(**p**_*I*_,**X**_*M*_) given by:

(6) $$  {\small{ \begin{aligned} \Phi(\mathbf{p}_{I},\mathbf{X}_{M})\,=\, \sqrt{\frac{1}{mK}\!\sum\limits_{k=1}^{K}(\mathbf{X}(t_{k};\!\mathbf{p}_{I})\,-\,\mathbf{X}_{M}(t_{k})\!)^{T}\!(\mathbf{X}(t_{k}; \mathbf{p}_{I})\,-\,\mathbf{X}_{M}(t_{k})\!)} \end{aligned} }}  $$

where *K* denotes the number of measurement time points, and **X**(*t*_*k*_;**p**_*I*_) is the simulated concentration prediction **X** at time *t*_*k*_. The calculation of *Φ*(**p**_*I*_,**X**_*M*_) involves several steps. First, given the values of **p**_*I*_, we evaluate the independent IF functions **η**_*I*,*k*_(**X**_*M*_,**p**_*I*_) according Eq. (5) using a modified Simpson’s rule (see Additional file 1: Section 1 and Figure S2 for more detail). Subsequently, we compute the dependent IFs **η**_*D*_(*t*_*k*_) using Eq. (4) and obtain the dependent parameter estimates as $\mathbf {p}_{D}^{*}=\arg \min _{\mathbf {p}_{D}}\Phi _{\textit {in}}(\mathbf {p}_{D},\mathbf {\eta }_{D},\mathbf {X}_{M})$ with the (inner) error function:

(7) $$  {\small{ \begin{aligned} &\Phi_{in}(\mathbf{p}_{D},\mathbf{\eta}_{D},\mathbf{X}_{M})\\ &\,\,=\! \sqrt{\frac{1}{mK}\!\sum\limits_{k=1}^{K}(\mathbf{\eta}_{D,k}(\mathbf{X}_{M},\mathbf{p}_{D})\,-\,\mathbf{\eta}_{D}(t_{k}))^{T} (\mathbf{\eta}_{D,k}(\mathbf{X}_{M},\mathbf{p}_{D})\,-\,\mathbf{\eta}_{D}(t_{k}))}. \end{aligned}}}  $$

where

(8) $$ \mathbf{\eta}_{D,k}(\mathbf{X}_{M},\mathbf{p}_{D})=\int_{0}^{t_{k}}\mathbf{v}_{D}(\mathbf{X}_{M},\mathbf{p}_{D})dt.  $$

The integration in Eq. (8) is also performed using a modified Simpson’s rule. When each of the parameters **p**_*D*_ appears only in one reaction rate equation, the minimization of *Φ*_*in*_ can be performed one reaction at a time. Finally, we compute **X**(*t*_*k*_;**p**_*I*_) either from **η**(**X**_*M*_,**p**) according to the integral form of the ODE model (see Eq. (2)) or by simulating the ODE model. In the case studies, the latter variant of the IFPE is labeled as IFPE-ODE to indicate that the calculation of the objective function requires solving the ODE model.

Finally, there maybe more than one way to appropriately partition the reactions into the independent and dependent subsets. Here, we use a few guidelines in selecting the independent subset. First and foremost, we select **η**_*I*_ such that the (pseudo-)inverse of **S**_*D*_ exists. As the computational cost of the nested optimization mainly scales with the parameter search space of the outer optimization, we therefore prefer **η**_*I*_ with fewer **p**_*I*_. Finally, we also consider prior information regarding the parameter values, and select the set of **η**_*I*_ with smaller ranges of **p**_*I*_ values.

## Results

Below we demonstrate the performance of the IFPE method on two case studies: a branched metabolic pathway [6] and a lin-log model of the glycolytic pathway in *L. lactis* [14]. In the case studies, we used CVODE subroutine from the package SUNDIALS (SUite of Nonlinear and DIfferential/ALgebraic equation Solvers) for the ODE integrations [15], with the option MaxNumSteps set to 5000. We performed the outer optimization in Eq. (6) using the enhanced Scatter Search (eSS) algorithm from SSmGO (Scatter Search for Matlab Global Optimization) toolbox [16-18], in which we terminated the parameter search when the objective function improved less than a relative tolerance of 10^−5^ for 50 successive iterations. Finally, for the inner optimization in Eq. (7), we employed the MATLAB subroutine *lsqcurvefit* with the trust region reflective option. In the outer and inner optimization, we enforced constraints on the parameter values to be within upper and lower bounds and to produce only positive IF values.

We compared the performance of the IFPE with the IPE [10]. In the IPE implementation, we first smoothened the time series data using piecewise polynomial spline fitting, and subsequently differentiated the spline functions to obtain estimates of *d***X**_*M*_(*t*)/*dt*. We also performed the outer optimization using the eSS algorithm with the same convergence criterion as in the IFPE implementation. However, for the inner optimization, we chose MATLAB function *quadprog* using interior-point-convex algorithm because in the two case studies below, the inner optimization problem constituted a quadratic programming problem. We implemented two variants of the IPE using different error functions for the outer optimization: the IPE-ODE method with the error function *Φ* in Eq. (6) and the IPE-slope method with the following error function:

(9) $$  {\small{ \begin{aligned} &\Phi_{IPE\ slope}(\mathbf{p}_{I},\mathbf{X}_{M})\\ &=\! \sqrt{\!\frac{1}{mK}\!\sum_{k=1}^{K}\!\left(\!\frac{d\mathbf{X}(t_{k};\mathbf{p}_{I})}{dt}\,-\,\frac{d\mathbf{X}_{M}(t_{k})}{dt}\!\right)^{\!T} \!\!\left(\!\frac{d\mathbf{X}(t_{k};\mathbf{p}_{I})}{dt}\,-\,\frac{d\mathbf{X}_{M}(t_{k})}{dt}\!\right)}. \end{aligned} }}  $$

Note that the IPE-slope method did not require any integration of the ODE model. We also enforced constraints on the parameter values by setting upper and lower bounds, and on the positivity on the reaction fluxes.

In addition to the IPE method, we further compared the IFPE to simultaneous parameter estimation (SPE) method. Here, we estimated the kinetic parameters by minimizing model prediction errors over all unknown parameters simultaneously. We also implemented two versions of the SPE method: the SPE-ODE method with the objective function in Eq. (6) and the SPE-slope method with the objective function in Eq. (9). We again used the eSS to find the global parameter solution using the same convergence criterion and parameter bounds as in the IFPE implementation.

### A generic branched pathway

**Figure 2:**
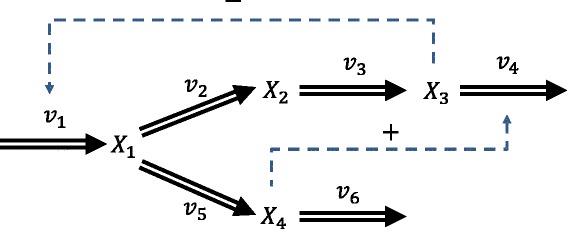
**Metabolic network of a generic branched pathway.** Double-line arrows indicate metabolic transformations and dashed arrows with plus or minus signs represent activation or inhibition, respectively.

The first case study comes from a generalized mass action (GMA) model of a generic branched pathway shown in Figure 2. Here, the rate equations follow the canonical power-law function:

(10) $$ v_{j}(\mathbf{X},\mathbf{p})=a_{j}\prod_{i}X_{i}^{g_{ji}},  $$

where *a*_*j*_ is the rate constant and *g*_*ji*_ is the kinetic order associated with the *i*-th metabolite in the *j*-th reaction. The generic branched pathway comprises four metabolites and six reactions, described by the ODE model: (11)

(12)

Using the parameters and initial conditions reported previously [6] (see Additional file 1: Section 2), we generated noise-free and noisy time-series concentration of all metabolites *X*_1_ to *X*_4_ (see Additional file 1: Figure S3 for the case of missing measurements). For the noisy dataset, we simulated five technical replicates under the same condition, assuming independent additive Gaussian noise with zero mean and 10% coefficient of variation. The time-series concentration data used in this example are available in Additional file 2. Here, we selected *v*_1_ and *v*_6_ as the independent reaction subset, leading to a square invertible **S**_*D*_ and the fewest **p**_*I*_ of 4 parameters. Furthermore, we constrained the rate constants to within [0,25] and the kinetic orders to within [0,2].

The median relative errors of the parameter estimates from the IFPE, IPE and SPE methods are given in Table 1. We performed the IPE and SPE-slope methods using several settings of piecewise spline fitting (see Additional file 1: Figure S4), where *s* is the number of piecewise sections and *o* is the degree of the polynomials. Comparing the outcomes of the IPE and SPE-slope methods using three different (*s*,*o*) combinations showed that the accuracy of the parameter estimates from these methods sensitively depends on the manner of which the time-series data are smoothen, especially for the IPE methods. We could generally obtain improved parameter accuracy by increasing the number of pieces and the degree of the polynomials. But, we urge caution when using more pieces and higher degree polynomials for spline fitting, as this may lead to data overfitting.

**Table 1:** **Comparison of median parameter errors for the branched pathway case study**

**Median parameter error** ^***a***^ ** (%)**	**Noise-free data** ^***b***^			**Noisy data** ^***c***^		
	(*s*,*o*)=(3,3)	(*s*,*o*)=(5,3)	(*s*,*o*)=(3,5)	(*s*,*o*)=(3,3)	(*s*,*o*)=(5,3)	(*s*,*o*)=(3,5)
SPE-slope	51.9	14.1	46.3	68.8 ± 19.4	81.2 ± 9.6	93.4 ± 7.4
IPE-slope	103	59.4	59.8	87.9 ± 4.3	68.6 ± 27.5	58.0 ± 21.6
IPE-ODE	50.0	19.7	7.74	90.9 ± 14.7	76.6 ± 35.6	71.1 ± 27.1
SPE-ODE	11.4 ^*d*^			37.9 ± 11.5		
IFPE	0.276			66.9 ± 32.5		
IFPE-ODE	0.746			70.0 ± 31.6		

^*a*^The median is taken over 13 parameters in the branched pathway model.

^*b*^For noise-free data, five independent runs were carried out. The median parameter error corresponds to the run with the lowest objective function value.

^*c*^For noisy data, the reported values are the mean ± standard deviation of five technical replicates of the data.

^*d*^Only three out of five repeated runs finished within 24 hours. The median parameter error is reported for the parameter estimate corresponding to the lowest objective function value among the three successful runs.

**Figure 3:**
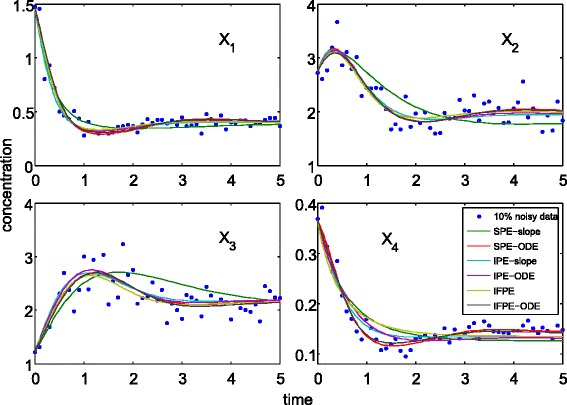
**Comparison of model predictions using the IPE, IFPE and SPE parameter estimates for the branched pathway case study.** The noisy data correspond to one set of the five technical replicates. For the IPE and SPE-slope estimates, the results correspond to (*s*,*o*)=(5,3).

The outcomes of the noise-free data showed that the IFPE methods could provide more accurate parameter estimates than the IPE and SPE methods. For the noisy dataset, the IFPE parameter estimates have similar accuracy to the IPE and SPE methods using the best data smoothing setting. However, in practice the optimal data smoothing setting is not known. Despite the differences in the accuracy of parameter estimates from the SPE, IPE and IFPE methods, Figure 3 shows that except for the SPE-slope, methods considered here could produce parameters with a reasonably good fit to the noisy concentration data (also see Additional file 1: Table S1 for more detail).

Tables 2 and 3 give the computational times and the number of eSS iterations for each of the estimation methods, respectively. In general, methods requiring the integration of fluxes and/or ODEs (i.e. IFPE, IFPE-ODE, IPE-ODE and SPE-ODE) took longer to converge than the rest. Here, the SPE-ODE was the slowest method as the optimization involved the entire parameter space **p** and the integration of ODEs. For GMA models with power-law rate equations, the inner optimization of the IPE simplified into a (log-)linear least square optimization, which could be solved much more efficiently than those in the IFPE. For this reason, the IPE-ODE method converged about twice faster than the IFPE-ODE despite requiring more eSS iterations. The IPE-slope method was the fastest among the methods considered as it did not require any integration. Interestingly, the parameter estimations using the noise-free dataset took longer to solve than those using the noisy dataset. In this regard, data noise has a smoothing effect on the objective function surface, and the low amount of noise enhanced the convergence to optimal solution [19]. Finally, we also observed high variability in repeated runs of eSS in the parameter estimation using noise-free data. Hence, for the noise-free results in Tables 1, 2 and 3, we reported the best parameter estimates corresponding to the lowest objective function value out of five repeated runs.

**Table 2:** **Comparison of CPU times for the branched pathway case study**

**CPU time** ^***a***^ ** (sec)**	**Noise-free data** ^***b***^			**Noisy data** ^***c***^		
	(*s*,*o*)=(3,3)	(*s*,*o*)=(5,3)	(*s*,*o*)=(3,5)	(*s*,*o*)=(3,3)	(*s*,*o*)=(5,3)	(*s*,*o*)=(3,5)
SPE-slope	933.84	1255.4	1182.6	207.7 ± 149.3	111.2 ± 70.5	40.2 ± 13.8
IPE-slope	106.48	104.55	185.38	96.8 ± 17.1	105.4 ± 16.0	102.9 ± 36.5
IPE-ODE	415.32	1380.1	1018.9	433.9 ± 70.6	456.4 ± 141.9	469.3 ± 174.4
SPE-ODE	14.8 hours ^*d*^			9002 ± 4839		
IFPE	1263			655.9 ± 198.5		
IFPE-ODE	2154			1023 ± 315		

^*a*^The CPU times were recorded using a workstation with Intel Xeon processor 3.33 GHz with 18 GB RAM.

^*b*^For noise-free data, five independent runs were carried out. The CPU time is reported for the run with the lowest objective function value.

^*c*^For noisy data, the reported values are the mean ± standard deviation of five technical replicates of the data.

^*d*^Only three our of five repeated runs finished within 24 hours. The CPU time corresponds to the run with the lowest objective function value among the three successful runs.

**Table 3:** **Comparison of the numbers of eSS iterations for the branched pathway case study**

**eSS iterations**	**Noise-free data** ^***a***^			**Noisy data** ^***b***^		
	(*s*,*o*)=(3,3)	(*s*,*o*)=(5,3)	(*s*,*o*)=(3,5)	(*s*,*o*)=(3,3)	(*s*,*o*)=(5,3)	(*s*,*o*)=(3,5)
SPE-slope	6730	9144	8636	1440 ± 1045	753.6 ± 490.7	259.0 ± 94.9
IPE-slope	63	65	143	66.2 ± 7.2	87.0 ± 18.6	92.4 ± 69.7
IPE-ODE	75	305	225	76.6 ± 17.8	83.8 ± 33.2	92.6 ± 43.3
SPE-ODE	3827 ^*c*^			787.8 ± 438.7		
IFPE	112			67.0 ± 13.1		
IFPE-ODE	156			70.2 ± 11.8		

^*a*^For noise-free data, five independent runs were carried out. The number of eSS iterations corresponds to the run with the lowest objective function value.

^*b*^For noisy data, the reported values are the mean ± standard deviation of five technical replicates of the data.

^*c*^Only three out of five repeated runs finished within 24 hours. The number of eSS iterations corresponds to the run with the lowest objective function value among the three successful runs.

In this example, there were more than one way to partition the fluxes into dependent and independent subsets, even when following the guidelines provided in the previous section. In order to investigate the sensitivity of the IFPE and IFPE-ODE performance with respect to the partitioning of the fluxes, we repeated the parameter estimation runs using five different dependent-independent sets, in which four runs involved the same number of independent parameters as above and one run involved a larger number of independent parameters (**v**_*I*_={*v*_1_,*v*_4_} had 5 independent parameters). The results are given in Tables S2–S5 in the Additional file 1, showing that the performance of the IFPE and IFPE-ODE is robust with respect to the flux partitioning.

### The glycolytic pathway in *Lactococcus lactis*

In the second case study, we consider the parameter estimation involving a lin-log (linear-logarithmic) modeling of the glycolytic pathway in *L. lactis* [14]. Here, the enzymatic reaction rate is expressed as a linear function of the logarithm of normalized concentrations [20] as follows:

(13) $$ \frac{v_{j}(\mathbf{X},\mathbf{p})}{{J_{j}^{0}}}=\frac{e_{j}}{{e_{j}^{0}}}\left(1+\sum_{i}\varepsilon_{ji}\ln\frac{X_{i}}{{X_{i}^{0}}}\right),  $$

where ${J_{j}^{0}}$ is the rate of the *j*-th reaction at the reference state, ${X_{i}^{0}}$ and ${e_{j}^{0}}$ denote the reference concentrations of the *i*-th metabolite and *j*-th enzyme, respectively, and *ε*_*ji*_ denotes the elasticity representing the influence of the *i*-th metabolite concentration on the *j*-th reaction rate. Lin-log models can be considered as an extension of metabolic control analysis (MCA) for dynamical systems, and have similar mathematical features to power-law rate equations.

**Figure 4:**
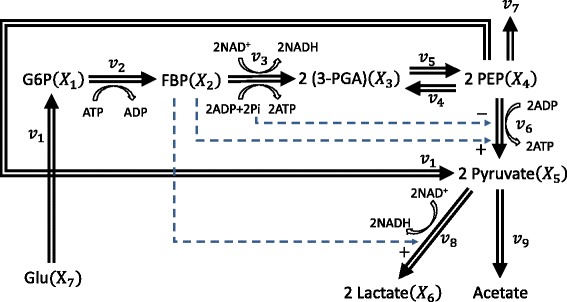
***L. lactis*** **glycolytic pathway.** Double-lined arrows show the flow of material, while dashed arrows with plus or minus signs represent activation or inhibition, respectively. Here, *v*_1_ describes the reaction flux of PEP + Glu → G6P + Pyruvate.

The metabolic pathway is shown in Figure 4, involving nine metabolites: glucose 6-phosphate (G6P) – *X*_1_, fructose 1, 6-biphosphate (FBP) – *X*_2_, 3-phosphoglycerate (3-PGA) – *X*_3_, phosphoenolpyruvate (PEP) – *X*_4_, Pyruvate – *X*_5_, Lactate – *X*_6_, external glucose (Glu) – *X*_7_, ATP – *X*_8_ and P_*i*_ – *X*_9_; and nine metabolic fluxes. The corresponding lin-log model is given by: (14)

(15)

The parameters of the lin-log model above have been simplified into *a*_*i*_’s and *g*_*ij*_’s, and thus do not necessarily have any direct physical interpretation. The experimental data consisted of time-series *in vivo* NMR measurements of the metabolites [21] (data taken from supplementary material of [14]).

**Figure 5:**
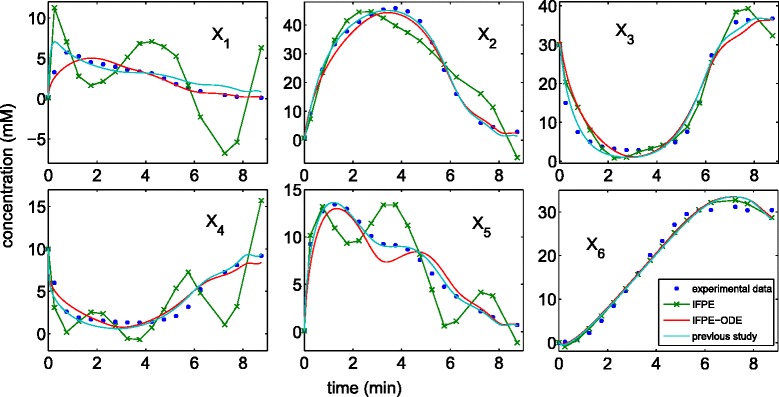
**Model prediction of concentration data.** For the IFPE without ODE integration, the concentration predictions were calculated from the integrated flux function at the given measurement time points using Eq. (2). For the IFPE-ODE, the concentration predictions were generated by integrating the ODE model. The concentration predictions of the previous study were generated by integrating the ODE model using the *lsqcurvefit* parameters in Table one of [14].

Following a previous parameter estimation case study of the above model, we considered only metabolite concentration data up to 10 minutes (see Figure 5), in order to avoid taking logarithms of zero concentration values [14]. We treated the external glucose, ATP and P_*i*_ (i.e. *X*_7_, *X*_8_ and *X*_9_) as off-line variables, using piecewise spline fitting of time-series concentration data with (*s*,*o*)=(6,4) (missing time-points were first linearly interpolated from the remaining data). We assigned *v*_5_, *v*_7_ and *v*_9_ as the independent fluxes to ensure an invertible **S**_*D*_ submatrix and the fewest independent parameters **p**_*I*_. Furthermore, we constrained all the parameters of the lin-log model to within [-500, 500]. For lin-log models, the inner optimization in the IFPE methods reduces to a linear least-square problem and thus can be performed very efficiently. In this case, we computed $\int lnX_{i}dt$ for each metabolite beforehand.

The parameter estimation of the lin-log model above has been shown to be extremely challenging. Using a deterministic optimization algorithm, a previous parameter estimation showed that the convergence to the optimal parameters depended strongly on prior information of the parameter values, which were used as initial parameter guess [14]. In particular, the only successful initial guess came from the parameter estimation for the GMA model of the same pathway. Here, the IPE method did not converge within a preset maximum time-limit of 24 hours. On the other hand, the two variants of the IFPE provided parameter estimates within the time-limit, which are summarized in Table 4. The IFPE method without integrating the ODE model was expectedly the quickest between the two IFPE variants, but the resulting fit to the data was rather poor, as shown in Figure 5. The slower IFPE-ODE method could produce parameter estimates that fit the time-series metabolite data as well as those from the previous study [14]. In contrast to the previous parameter estimation, the IFPE-ODE however does not require any prior information on parameter values, aside from the parameter bounds.

**Table 4:** **Performance comparison for the lin-log modeling of** ***L. lactis*** **glycolytic pathway**

	**CPU Time** ^***a***^ ** (sec)**	***Φ***	**eSS Iterations**
IFPE	152.7	3.362	73
IFPE-ODE	3354	1.723	133

^*a*^The CPU time was recorded using a workstation with Intel Xeon processor 3.33 GHz with 18 GB RAM.

## Discussion

The estimation of kinetic parameters of ODE models represents an active research area in systems biology. The main challenges in this topic involve the ill-posedness of the estimation problem such that many indistinguishable solutions exist [4], as well as the high computational cost in solving the associated multidimensional global optimization problem. In this work, we present a new parameter estimation method, called integrated flux parameter estimation, based on an incremental approach using integrated reaction fluxes. In the IFPE, we formulate the parameter estimation as a nested optimization problem, in which we decouple the parameter estimation into outer and inner optimization over independent and dependent reaction subspaces, respectively. In comparison to standard estimation strategies, the outer and inner optimizations of the IFPE method involve smaller parameter dimension, translating to faster convergence and computational time. As demonstrated in the case studies, the nested optimization strategy could offer a significant improvement in computational speed over the standard simultaneous estimation.

The IFPE offers a couple advantages over a previous method, the IPE, which uses a similar nested optimization. One weakness of the IPE method is that the parameter accuracy sensitively depends on the data smoothing procedure, as demonstrated in Table 1. Unfortunately, the optimal data smoothing setting for a given dataset, one that leads to the most accurate parameter estimates, is usually not known. In contrast, the IFPE does not require any time-series data smoothing and differentiation, and is therefore not affected by the issue above. In addition, as the IFs are directly estimated from time-series data, the IFPE can provide much lower parameter bias than the IPE. The advantages of the IFPE over the IPE come at the cost of increased computational time due to the numerical integration of reaction flux equations. We note that in the case studies, the increase in the computational cost was reasonably low, where the IFPE methods were typically 1.5 to 2 times slower than the IPE method with ODE integrations (i.e. IPE-ODE). In some cases, such as in the parameter estimation of lin-log models, the IFPE however could offer better computational performance than the IPE by taking advantage of the structure of the reaction flux functions.

In the first example (the branched pathway model), we tested the performance of the two variants of IFPE using a GMA model with *in silico* noise-free and noisy data. Here, we used the same model equations in the data generation and the parameter estimation. Thus, this example represented an idealized parameter estimation case study, where the only unknown information in the model was the parameter values. The parameter estimates from noise-free data indicated that both IFPE variants could produce nearly unbiased estimates with median parameter errors of less than 1%. The high parameter accuracy using noise-free data suggested that the parameters are *a priori* identifiable. Noise in data expectedly led to less accurate parameter estimates, not only for the IFPE methods, but also for the other estimation methods. In the second example, we applied the IFPE to another popular class of metabolic network models, namely lin-log kinetic model. The dataset in the second case study was less dense than that in the first example. In addition, the resulting data fitting also appeared worse than that in the first example. However, the mismatch between model prediction and concentration data depends not only on the accuracy of the parameter values, but also on how well model equations approximate the metabolic reactions. In this regard, the lin-log model in Eqs. 14-15 has difficulty in describing the transient behavior of *L. lactis* metabolism. This issue is not surprising as the lin-log kinetics are derived from thermodynamics concepts and are in principle valid only around steady state (or reference state) [20]. As shown in Figure 5, the IFPE method could fit the concentration data as well as the simultaneous estimation approach using an optimized initial parameter guess.

The computational cost of the IFPE is a product of the number of iterations in the outer optimization and the cost of each iteration. In general, the computational complexity of finding global optimal solution(s) is expected to increase exponentially with the dimension of the search space [22]. Assuming that each flux function has roughly a fixed number of unknown parameters, we thus expect that the complexity of the outer optimization of the IFPE scales exponentially with the number of independent reactions. Meanwhile, the cost per iteration is associated with the integration of reaction flux functions, the inner optimization, and in the case of IFPE-ODE, the integration of ODE model. When kinetic parameters are not shared among reaction rate equations, the inner optimization could be performed one-reaction-at-a-time and thus its complexity should increase linearly with the number of dependent fluxes. Meanwhile, the ODE integrations can slow down the parameter estimation significantly, especially when the ODE model is stiff.

Here, we have used the eSS global optimization algorithm, which is a population-based metaheuristic method combining scatter search and local deterministic optimizations. In comparison to deterministic global optimization algorithms, metaheuristic methods have better computational scalability. But, these methods lack rigorous guarantee in convergence to the global optimal solution, and repeated runs of the algorithm may not necessarily converge to the same solution. In the eSS algorithm, we track a population of parameter solutions, which is updated at every iterations. As the recommended size of the population increases linearly with the dimension of the search space, the computational cost per iteration should also increase linearly with the dimension of **p**_*I*_. Unfortunately, it is difficult to predict how the convergence rate of eSS would scale with the dimension of **p**_*I*_. This is because the convergence rate of eSS or any numerical optimization algorithm depends not only on the problem size but also on the topology of the search space (J. Banga, private communication).

## Conclusions

The estimation of ODE model parameters from time-series data is often the bottlenecking step in biological system modeling. In this work, we develop a reliable and efficient parameter estimation method, called integrated flux parameter estimation, for ODE models having more reactions than (measured) species. Such ODE models are common in biology as cellular species often participate in more than one reaction and concentration measurements are available only for a fraction of the species. The IFPE method relies on the integral form of the ODE model, based on which one can compute the integrated fluxes directly from time-series concentration data. Here, the reactions are partitioned into independent and dependent subsets such that the dependent IFs can be uniquely determined from the independent IFs. We formulate the parameter estimation as a nested optimization problem, where the outer optimization involves the minimization of model prediction errors over the independent parameters (i.e. parameters appearing in the independent reaction rates), and the inner optimization involves the minimization of IF prediction errors over the dependent parameters (i.e. parameters appearing in the dependent reaction rates). Using two case studies comprising a GMA model of branched metabolic pathway and a lin-log model of *L. lactis* glycolytic pathway, we show that the IFPE can produce parameter estimates with a low bias and in much faster computational times than standard parameter estimation method based on simultaneous approach. In comparison to a previously published nested estimation method, the IFPE does not require any smoothing and differentiation of noisy time-series data. These advantages come at the cost of a small increase in the computational times. The IFPE and IPE are available through a MATLAB user interface called REDEMPTION (Reduced Dimension Ensemble Modeling and Parameter Estimation) at http://www.cabsel.ethz.ch/tools/redemption, or upon request.

## Additional files

Additional file 1
**Supplementary to parameter estimation of dynamic biological network models using integrated fluxes.** The additional file contains the flowchart of integrated flux parameter estimation (IFPE) methods with unmeasured concentrations, the modified Simpson’s rule used in the calculation of IFs, the parameters and initial conditions used to generate time-series data in the branched pathway case study, the smoothened time-series concentration figure using different piecewise spline-fitting parameters, the comparison of concentration errors *Φ*, the parameter estimation results using noise-free data without the concentration measurements of *X*_3_, and the IFPE performance with different selections of independent fluxes.

Additional file 2
**Time-series Concentration Data for the Branched Pathway Case Study.** The additional file contains *in silico* noise-free and noisy dataset used in the case study of branched metabolic pathway.
