# Supplementary material for: Parameter estimation of dynamic biological network models using integrated fluxes
Source: BMC Syst Biol. 2014 Nov 18;8:127. doi: 10.1186/s12918-014-0127-x (PMC4241227; doi:10.1186/s12918-014-0127-x)
Supplement: Additional file 1 — Supplementary to parameter estimation of dynamic biological network models using integrated fluxes. The additional file contains the flowchart of integrated flux parameter estimation (IFPE) methods with unmeasured concentrations, the modified Simpson’s rule used in the calculation of IFs, the parameters and initial conditions used to generate time-series data in the branched pathway case study, the smoothened time-series concentration figure using different piecewise spline-fitting parameters, the comparison of concentration errors Φ, the parameter estimation results using noise-free data without the concentration measurements of X 3, and the IFPE performance with different selections of independent fluxes. [file 12918_2014_127_MOESM1_ESM.pdf]

METHODOLOGY ARTICLE

Supplementary to parameter estimation of dynamic biological network models using integrated fluxes

Yang Liu and Rudiyanto Gunawan*

*Correspondence:
rudi.gunawan@chem.ethz.ch
Institute for Chemical and Bioengineering, ETH Zurich, Vladimir-Perlog-Weg 1, 8093 Zurich, Switzerland
Full list of author information is available at the end of the article

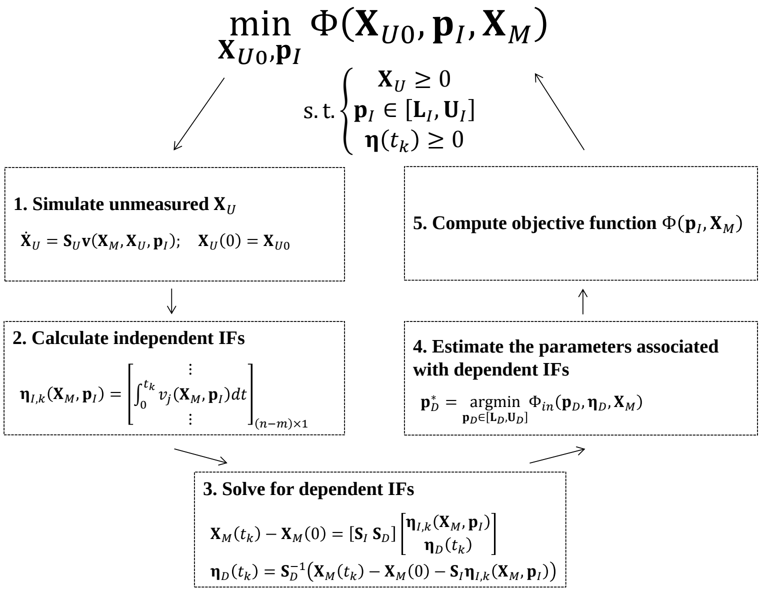

Figure S1 Flowchart of integrated flux parameter estimation (IFPE) with unmeasured concentrations. These unmeasured species are denoted by $\mathbf{X}_U$. All reactions and parameters appearing in $\dot{\mathbf{X}}_U$ are selected as independent fluxes and parameters. The first step in calculating Φ involves simulating unmeasured concentration $\mathbf{X}_U$ by solving ODEs $\dot{\mathbf{X}}_U = \mathbf{S}_U\mathbf{v}(\mathbf{X}_M, \mathbf{X}_U, \mathbf{p}_I)$, where the measured concentrations $\mathbf{X}_M$ are treated as external input variables. The subsequent steps are the same as in the IFPE methods without unmeasured concentrations. An example using the branched pathway model is shown in Figure S3.

1 Modified Simpson's rule

We performed the integration of flux function $\eta(\mathbf{X}, \mathbf{p}) = \int_0^t \mathbf{v}(\mathbf{X}, \mathbf{p})dt$ using the following modified Simpson's quadrature rule:

$$\int_{t_1}^{t_2} f(t)dt \approx \frac{t_2 - t_1}{6}\left[\frac{2+3\beta}{1+\beta}f(t_1) + \frac{1+3\beta}{\beta}f(t_2) - \frac{1}{\beta(1+\beta)}f(t_3)\right], \tag{1}$$

where $\beta = (t_3 - t_2)/(t_2 - t_1)$. The quadrature function above was derived from the ordinary Simpson's rule, by calculating the shaded area of the quadratic polynomial determined by three points $(t_1, f(t_1))$, $(t_2, f(t_2))$, and $(t_3, f(t_3))$ as illustrated

in Figure S2. In the special case that the time-series data are taken at equally distributed time points, the quadrature function above reduces to (by setting $\beta = 1$):

$$\int_{t_1}^{t_2} f(t)dt \approx \frac{dt}{12}\left[5f(t_1) + 8f(t_2) - f(t_3)\right]. \tag{2}$$

To calculate the integral between last two time points, the area between t_{N-1} and t_N under the curve of the quadratic polynomial is

$$\int_{t_{N-1}}^{t_N} f(t)dt \approx \frac{t_N - t_{N-1}}{6}\left[-\frac{1}{\beta'(1+\beta')}f(t_{N-2}) + \frac{1+3\beta'}{\beta'}f(t_{N-1}) + \frac{2+3\beta'}{1+\beta'}f(t_N)\right], \tag{3}$$

where $\beta' = (t_{N-1} - t_{N-2})/(t_N - t_{N-1})$.

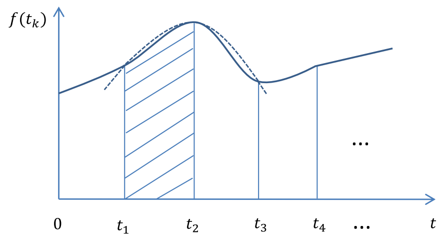

Figure S2 Modified Simpson's rule

2 Supplementary information for the branched pathway case study

We generated the *in silico* data by simulating the ODE model in Eqs. (11) and (12) using the parameter values: $a_1 = 12$, $g_{13} = 0.8$, $a_2 = 8$, $g_{21} = 0.5$, $a_3 = 3$, $g_{32} = 0.75$, $a_4 = 5$, $g_{43} = 0.5$, $g_{44} = 0.2$, $a_5 = 2$, $g_{51} = 0.5$, $a_6 = 6$, $g_{64} = 0.8$, and the initial conditions:

$$\begin{bmatrix} X_1(0) \\ X_2(0) \\ X_3(0) \\ X_4(0) \end{bmatrix} = \begin{bmatrix} 1.4 \\ 2.7 \\ 1.2 \\ 0.4 \end{bmatrix}.$$

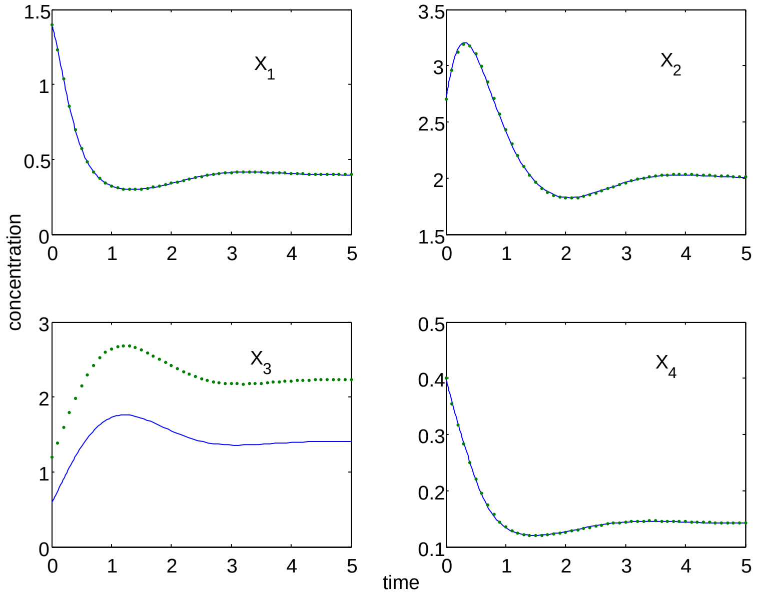

Figure S3 Parameter estimation of the branched pathway model using noise-free data without the concentration measurements of X_3 using the IFPE without ODE integration. The solid lines and dots represent the model prediction and the true concentrations, respectively.

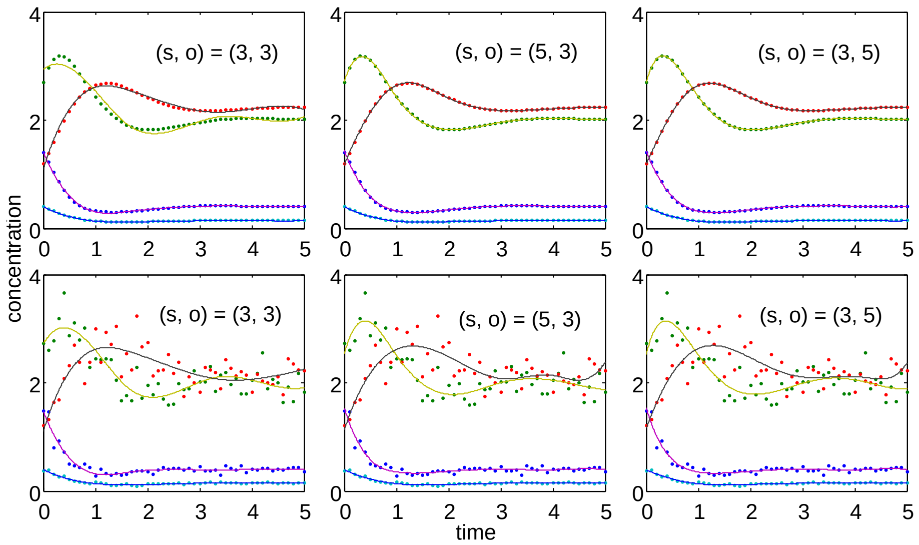

Figure S4 Smoothened time-series concentration data using different piecewise spline-fitting parameters for the branched pathway case study. The parameters s and o indicate the number of pieces and the degree of polynomials, respectively. The plots in first row show noise-free data, while the plots in second row show one noisy dataset (out of five technical replicates) with 10% coefficient of variation of Gaussian noise.

Table S1 Comparison of concentration errors Φ for the branched pathway case study

Concentration error	Noise-free data[a]			Noisy data[b]		
	$(s,o)=(3,3)$	$(s,o)=(5,3)$	$(s,o)=(3,5)$	$(s,o)=(3,3)$	$(s,o)=(5,3)$	$(s,o)=(3,5)$
SPE-slope	6.18×10^{-2}	2.27×10^{-2}	1.95×10^{-2}	0.280 ± 0.165	0.217 ± 0.022	0.227 ± 0.024
IPE-slope	6.03×10^{-2}	1.73×10^{-2}	1.40×10^{-2}	0.183 ± 0.031	0.179 ± 0.021	0.178 ± 0.018
IPE-ODE	2.37×10^{-2}	7.44×10^{-3}	4.12×10^{-3}	0.162 ± 0.012	0.160 ± 0.011	0.160 ± 0.011
SPE-ODE		2.48×10^{-3}[c]			0.157 ± 0.014	
IFPE		2.83×10^{-4}			0.161 ± 0.014	
IFPE-ODE		1.65×10^{-4}			0.157 ± 0.014	

a. For noise-free data, five independent runs were carried out. The concentration error is reported for the run with the lowest objective function value.
b. For noisy data, the reported values are the mean ± standard deviation of five technical replicates of the data.
c. Only three out of five repeated runs finished within 24 hours. The concentration error is reported for the run with the lowest objective function value among the three successful runs.

Table S2 Comparison of median parameter errors for the branched pathway case study with different partitioning of independent and dependent flux set.

Median parameter error[a] (%)	Noise-free data[b]		Noisy data[c]	
Independent fluxes	IFPE	IFPE-ODE	IFPE	IFPE-ODE
$\{V_1, V_6\}$[d]	0.276	0.746	66.9 ± 32.5	70.0 ± 31.6
$\{V_1, V_5\}$	0.293	0.734	64.0 ± 33.0	50.6 ± 26.9
$\{V_2, V_6\}$	0.270	1.00	68.6 ± 32.2	70.7 ± 27.7
$\{V_1, V_3\}$	0.262	0.755	59.1 ± 26.4	60.6 ± 15.0
$\{V_2, V_5\}$	0.281	0.975	66.1 ± 27.9	66.8 ± 33.8
$\{V_1, V_4\}$	0.238	0.839	60.3 ± 29.6	68.5 ± 20.9

a. The median is taken over 13 parameters in the branched pathway model.
b. For noise-free data, five independent runs were carried out. The median parameter error corresponds to the run with the lowest objective function value.
c. For noisy data, the reported values are the mean ± standard deviation of five technical replicates of the data.
d. Independent flux combination used in the main text.

Table S3 Comparison of CPU times for the branched pathway case study with different partitioning of independent and dependent flux set.

CPU time[a] (sec)	Noise-free data[b]		Noisy data[c]	
Independent fluxes	IFPE	IFPE-ODE	IFPE	IFPE-ODE
$\{V_1, V_6\}$[d]	1263	2154	655.9 ± 198.5	1023 ± 315
$\{V_1, V_5\}$	1806.8	2912.3	677.4 ± 205.0	956.6 ± 231.0
$\{V_2, V_6\}$	1995.7	1775.4	1190.1 ± 332.6	1809.9 ± 589.4
$\{V_1, V_3\}$	1152.9	2399.7	562.6 ± 334.2	1529.9 ± 656.5
$\{V_2, V_5\}$	2961.8	3659.2	1285.9 ± 141.1	2055.4 ± 392.8
$\{V_1, V_4\}$	1527.0	3267.9	1067.8 ± 890.5	1241.8 ± 299.0

a. The CPU times were recorded using a workstation with Intel Xeon processor 3.33GHz with 18GB RAM.
b. For noise-free data, five independent runs were carried out. The CPU time is reported for the run with the lowest objective function value.
c. For noisy data, the reported values are the mean ± standard deviation of five technical replicates of the data.
d. Independent flux combination used in the main text.

Table S4 Comparison of the number of eSS iterations for the branched pathway case study with different partitioning of independent and dependent flux set.

eSS iterations	Noise-free data[a]		Noisy data[b]	
Independent fluxes	IFPE	IFPE-ODE	IFPE	IFPE-ODE
$\{V_1, V_6\}$[c]	112	156	67.0 ± 13.1	70.2 ± 11.8
$\{V_1, V_5\}$	158	201	64.2 ± 5.9	66.8 ± 2.9
$\{V_2, V_6\}$	147	86	92.0 ± 35.8	96.8 ± 41.4
$\{V_1, V_3\}$	169	241	113.0 ± 61.0	151.6 ± 69.2
$\{V_2, V_5\}$	194	160	63.4 ± 2.6	80.6 ± 31.6
$\{V_1, V_4\}$	162	252	154.0 ± 75.1	78.6 ± 15.2

a. For noise-free data, five independent runs were carried out. The number of eSS iterations corresponds to the run with the lowest objective function value.
b. For noisy data, the reported values are the mean ± standard deviation of five technical replicates of the data.
c. Independent flux combination used in the main text.

Table S5 Comparison of concentration error Φ for the branched pathway case study with different partitioning of independent and dependent flux set.

concentration error	Noise-free data[a]		Noisy data[b]	
Independent fluxes	IFPE	IFPE-ODE	IFPE	IFPE-ODE
$\{V_1, V_6\}^c$	2.83×10^{-4}	1.65×10^{-4}	0.161 ± 0.014	0.157 ± 0.014
$\{V_1, V_5\}$	2.89×10^{-4}	1.71×10^{-4}	0.159 ± 0.015	0.158 ± 0.014
$\{V_2, V_6\}$	2.86×10^{-4}	1.81×10^{-4}	0.161 ± 0.014	0.159 ± 0.013
$\{V_1, V_3\}$	2.83×10^{-4}	1.68×10^{-4}	0.160 ± 0.016	0.157 ± 0.014
$\{V_2, V_5\}$	2.85×10^{-4}	1.85×10^{-4}	0.160 ± 0.014	0.159 ± 0.013
$\{V_1, V_4\}$	2.80×10^{-4}	1.54×10^{-4}	0.161 ± 0.015	0.157 ± 0.014

a. For noise-free data, five independent runs were carried out. The concentration error is reported for the run with the lowest objective function value.
b. For noisy data, the reported values are the mean ± standard deviation of five technical replicates of the data.
c. Independent flux combination used in the main text.
